# Ingratitude to Parents

**Published:** 1878-08

**Authors:** 


**Ingratitude to Parents.**—There was once a father who gave up every thing to his children—his house, his fields, and goods—and expected that for this his children would support him. But after he had been some time with his son, the latter grew tired of him, and said to him: "Father, I have had a son born to me this night, and there, where your arm-chair stands, the cradle must come. Will you not, perhaps, go to my brother, who has a larger room?'

After he had been some time with the second son, he also grew tired of him, and said: "Father, you like a warm room, and that hurts my head. Won't you go to my brother, the baker?" The father went, and after he had been some time with the third son, he also found him troublesome, and said to him: "Father, the people run in and out here all day, as if it were a pigeon-house, and you can not have your noonday sleep. Would you not be better off at my sister Kate's, near the town-wall?"

The old man remarked how the wind blew, and said to himself: "Yes, I will do so; I will go and try it with my daughter. Women have softer hearts." But after he had spent some time with his daughter, she grew weary of him, and said she was always so fearful when her father went to church, or any where else, and was obliged to descend the steep stairs, and at her sister Elizabeth's there were no stairs to descend, as she lived on the ground-floor.

For the sake of peace the old man assented, and went to his other daughter. But after some time she, too, was tired of him, and told him, by a third person, that her house near the water was too damp for a man who suffered with gout, and her sister, the grave-digger's wife, at St. John's, had much drier lodgings. The old man himself thought she was right, and went outside the gate to his youngest daughter, Helen. But after he had been three days with her, her little son said to his grandfather: "Mother said yesterday to cousin Elizabeth, that there was no better chamber for you than such a one as father digs." These words broke the old man's heart, so he sank back in his chair and died.—*Martin Luther.*

